# Design factors for determining the radula shape of Euhadra Peliomphala

**DOI:** 10.1038/s41598-018-36397-x

**Published:** 2019-01-24

**Authors:** Satoshi Miura, Rikako Saito, Victor Parque, Tomoyuki Miyashita

**Affiliations:** Department of the Modern Mechanical Engineering, Waseda University, 3-4-1, Okubo, Shinjuku, Tokyo Japan

## Abstract

Biomimetics present useful ideas for various product designs. However, most biomimetics only mimic the features of living organisms. It has not been clarified how a given shape is attained through natural selection. This paper presents the design factors that optimize the radula shape of Euhadra peliomphala. Clarifying the important design factors would help designers in solving several problems simultaneously in order to adapt to complicated and multi-functionalized design mechanisms. We measured the radula of Euhadra peliomphala by using a microscope and modeled the grinding/cutting force using the finite element analysis (FEA). We reproduced the natural selection using multi-objective genetic algorithm (MOGA). We compared the solutions when optimizing the radula shape using objective functions of each combination of stress, cutting force, abrasion, or volume. The results show that the solution obtained through two-objective optimization with stress and cutting force was the closest to the actual radula shape.

## Introduction

Biomimetics mimic the processes and structures that living organisms adopt to survive in the environment for various product designs^1–3^. Otto Schmitt proposed “biomimetics” for the first time to mimic the signal processing of a neural network and develop an electric circuit for eliminating noise and converting it into a rectangular wave called “Schmitt trigger”^4,5^. There are several studies on biomimetics. Velcro was developed to mimic burdock seeds^6^. Adhesive tape was developed to mimic the Van der Waals forces on the skin of a Gecko’s feet that support the body weight of the Gecko^7^. TEIJIN Inc. developed rain wear with ultra-hydrophobicity by mimicking lotus leaves^8,9^. LIXIL developed self-cleaning building materials to mimic the biological tissue of snails^10^. Furthermore, Jin studied the nanomechanical properties of dragonfly wings (Anax parthenope)^11^. Micro air vehicles were developed to implement biomimetic design^12^. However, these studies have only mimicked the features of shapes, and have not discussed the factors behind shape designs.

In fact, in optimization in manufacturing, there are requirements and restrictions in terms of various performances, manufacturing cost, and design variables within a certain range like dimensions. An optimization method is used to obtain the most appropriate solution among the given conditions. In optimization, the relationship between the design variables and the requirement item is shown as the objective function. The solution is derived to maximize or minimize the objective function within the range of the design variables. For actual product design, the satisfaction of multi-objective function is required. For example, quality, cost, delivery (QCD) is the main issue for designers^13–15^. In addition, a universal design is regarded important^16,17^. Furthermore, reduction of power consumption^18^ and weight^19^ is also indispensable for product design. Therefore, designers require a method to solve multiple problems simultaneously to adapt to complicated and multi-functionalized design mechanism^20–22^. Conventionally, designers have solved problems depending on extensive experience and intuition^23,24^. However, experience and intuition vary among individuals and this will lead to different solutions and will not necessarily result in an optimal solution^25^. It is necessary to clarify a method to satisfy multiple design indicators. Wolfram was the first to suggest that the shapes of many living organisms can be given as mathematically optimal solutions^26^. However, conventionally, there are few studies on the factors determining the shape of living organisms. Berthe studied the frictional properties of snake skins to analyze the detailed relationship between biological and frictional structure^27^, but did not clarify which factors helped in determining shape.

**Figure 1:**
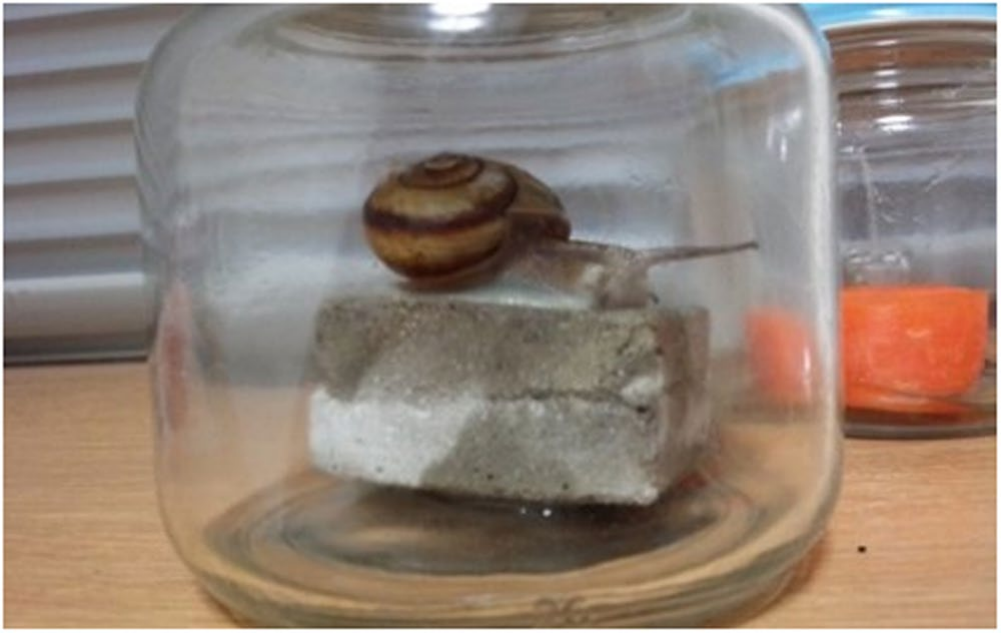
Euhadra peliomphala.

**Figure 2:**
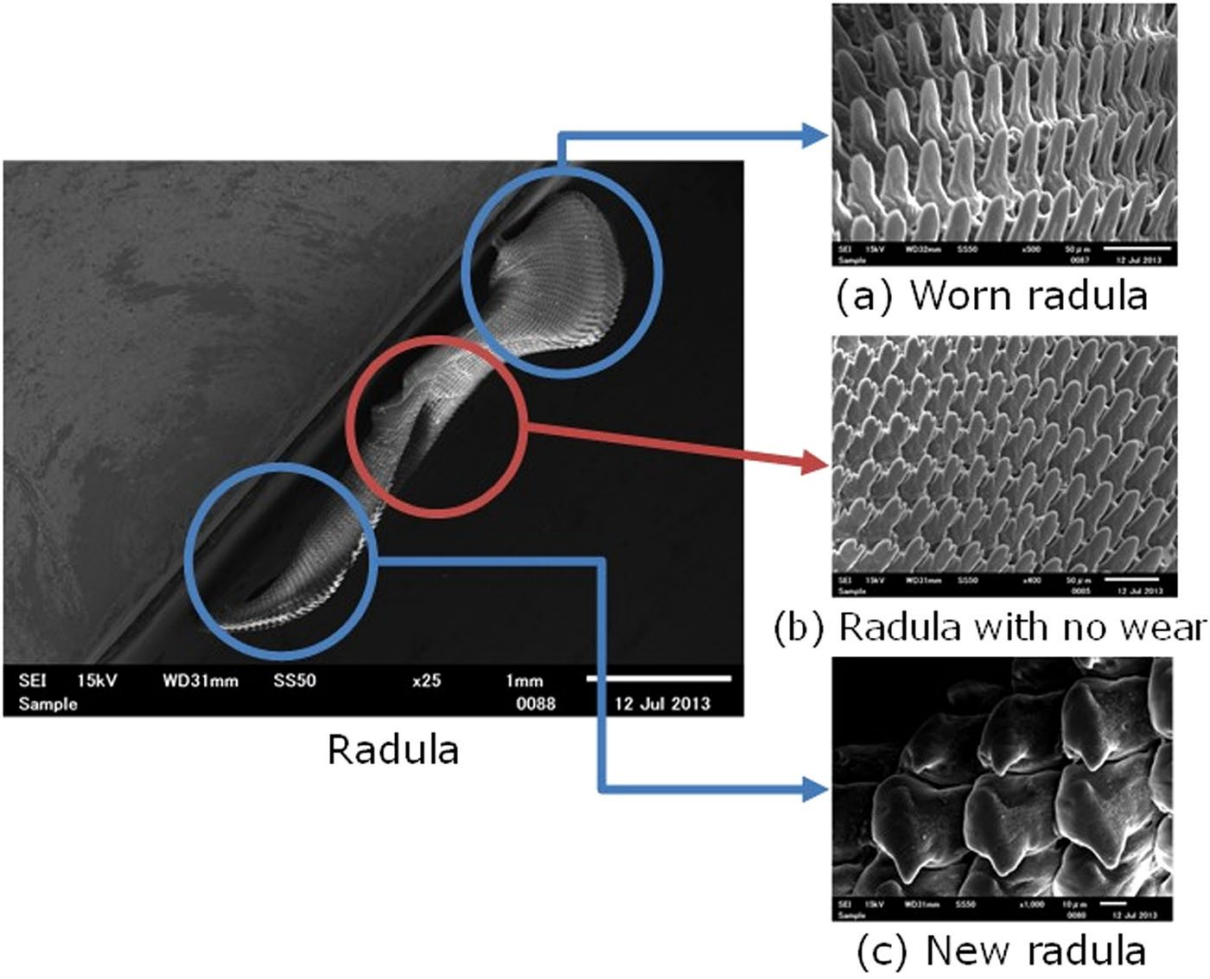
Observation of Radula. The worn radula is sharp and pointed, as shown in (**a**), but radula with no wear and new radula are short and rounded, as shown in (**b**,**c**), respectively. Euhadra peliomphala eats concrete blocks to consume calcium and maintain its shell. The radula is made of calcium carbonate, chitin, and protein. It is difficult to consume calcium, and thus, the shape of the radula is advantageous for grinding preferably. The gap of each radula is 26.0–40.0 [μm].

In this paper, we aim to clarify the important indicators for an optimal design by simulating natural selection based on biomimetics. Most biomimetics have heuristically mimicked shapes^28,29^. We examine which design indicators are used from the shapes of living organisms by simulating the natural selection with the multi-objective genetic algorithm (MOGA). We simulate the dynamic behavior and shape of living organisms by using CAE and a multi-objective optimization method to examine what factors optimize the shape. In this study, we selected a land snail, Euhadra peliomphala as a model case, as shown in Fig. 1. Euhadra peliomphala can mostly be found in Kanto district and eat plants and concrete^30,31^. The tongue of Euhadra peliomphala is called the “radula” as shown in Fig. 2, and it consists of regularly aligned projections^32^. Euhadra peliomphala uses the radula to scrap hard concrete and eat^33^. The lamellar microstructure on Euhadra peliomphala’s radula is consisted of the basic building blocks like the Pectinidae, Aragonite and Red Abalone^34–36^. The old radula at the tip is replaced with new radula generated from the radula sac in a manner similar to that of human nails^37^. In natural selection, radula would have been optimized to satisfy the multi-objective. The candidates for objective function are the following four. One is minimizing the cutting force of the radula. Next is minimizing the stress on the radula. Three is minimizing the radula volume because calcium contained in radula is difficult to ingest. Four is minimizing the amount of abrasion per unit time during grinding of the radula. We hypothesized that the radula would have been optimized by any of these four objective functions, or a combination thereof. We simulate the natural selection by MOGA and reveal the shape of the radula so that one or combination of these four objective functions minimizes. We clarified that the objective function having solution of radula shape closest to the actual radula shape is emphasized by natural selection. The factors that optimize the shape of the radula would be useful in designing a scraping mechanism such as a lathe.

## Results

### Grinding force/cutting force

We observed the eating trace of Euhadra peliomphala to decide grinding and cutting forces, as listed in Tables 1–3. Similar to the determination of Cp, we compared the movement of the radula and the grinding/cutting force model, as summarized in Tables 4 and 5.

**Table 1:** Eating traces of Euhadra peliomphala.

Euhadra peliomphala A		Euhadra peliomphala B	
Grinding radius of radula *D*/2 [mm]	Cutting depth Δ [mm]	Grinding radius of radula D/2 [mm]	Cutting depth Δ [mm]
0.322	0.655	0.331	0.614
0.244	0.629	0.274	0.740
0.199	0.691	0.152	0.801
0.169	0.514	0.258	1.220
0.244	0.755	0.199	0.652
0.236	0.622	0.243	0.623

**Table 2:** Parameters of Euhadra peliomphala A.

Radula moving speed V[mm/s]	Feeding speed v mm/s	Grinding width b [mm]	Effective tooth number N
3.04	0.1	1.0	648
2.30	0.1	1.0	648
1.88	0.1	1.0	648
1.59	0.1	1.0	648
2.30	0.1	1.0	648
2.22	0.1	1.0	648

**Table 3:** Parameters of Euhadra peliomphala B.

Radula moving speed V[mm/s]	Feeding speed v mm/s	Grinding width b [mm]	Effective tooth number N
3.12	0.1	0.8	444
2.58	0.1	0.8	444
1.43	0.1	0.8	444
2.43	0.1	0.8	444
1.88	0.1	0.8	444
2.29	0.1	0.8	444

**Table 4:** Grinding/cutting force of Euhadra peliomphala A.

Tangential grinding force Ft × 10^−2^ [N]	Vertical grinding force Fn × 10^−3^ [N]	Tangential cutting force *f*_*t*_ × 10^−5^ [N]	Vertical cutting force *f*_*t*_ × 10^−5^ [N]
1.36	5.52	2.10	0.851
1.79	7.53	2.76	1.16
2.40	9.23	3.70	1.42
2.15	10.87	3.32	1.68
2.12	7.53	3.27	1.16
1.96	7.53	3.03	1.16
1.96	8.04	3.03	1.24

**Table 5:** Grinding/cutting force of Euhadra peliomphala B.

Tangential grinding force Ft × 10^−2^ [N]	Tangential grinding force Ft × 10^−2^ [N]	Tangential grinding force Ft × 10^−2^ [N]	Tangential grinding force Ft × 10^−2^ [N]
1.03	4.44	2.33	0.999
1.48	5.37	3.34	1.21
2.87	9.67	6.47	2.18
2.52	5.70	5.68	1.28
1.81	7.39	4.08	1.66
1.94	6.51	4.38	1.47

### Response surface of radula shape optimization

We compare the following objective functions as following:

Objective function I: Minimize cutting force.

Objective function II: Minimize radula stress.

Objective function III: Minimize radula volume (because it is difficult to consume calcium in radula).

Objective function IV: Minimize abrasion amount per unit time by radula grinding.

**Figure 3:**
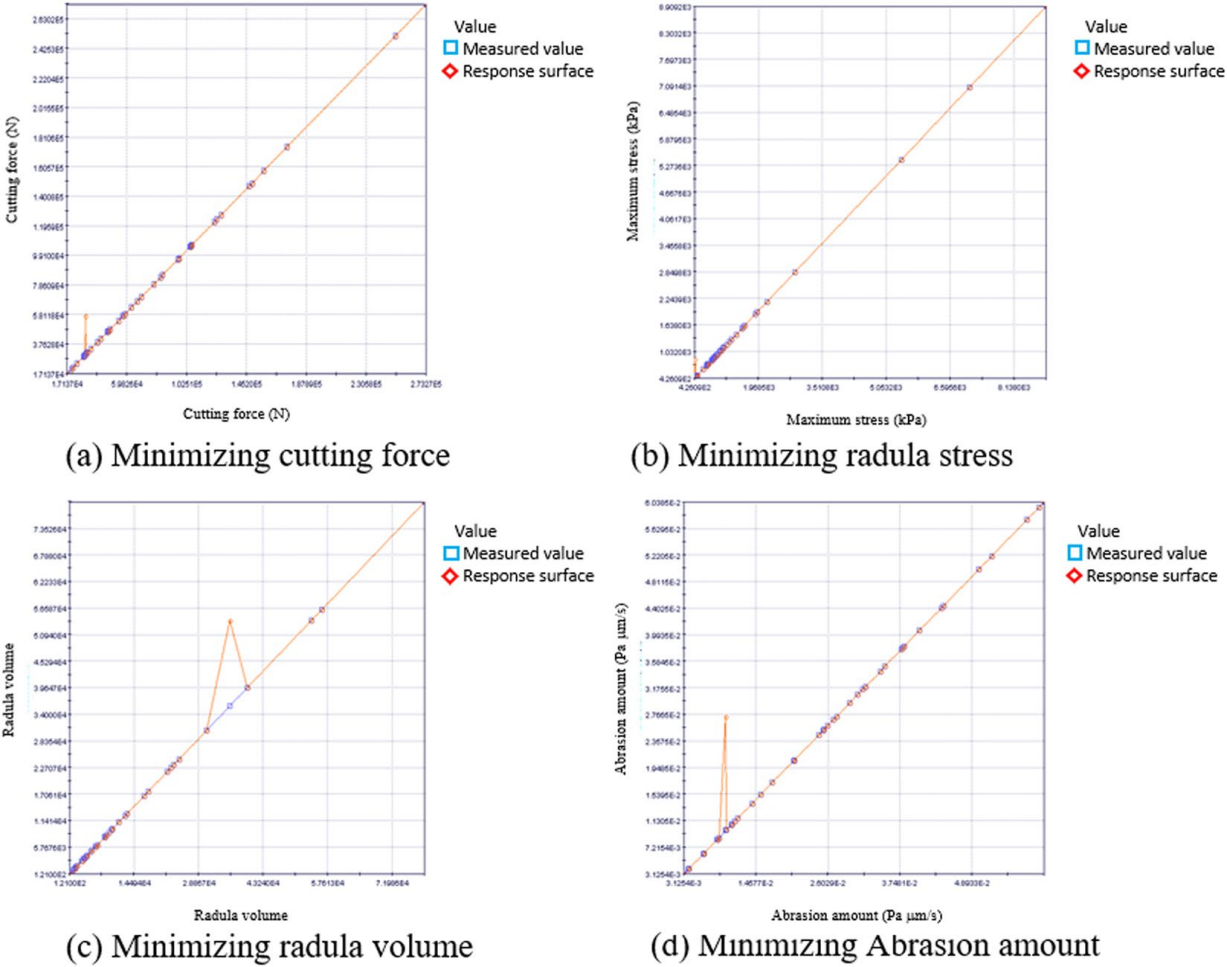
Error between the value on each objective function response surface and measured value.

We made the response surfaces between each objective function I-IV and each radula parameters such as the rake angle, round, tooth angle, and tooth height. From these response surfaces, we clarified the error between the measured value and the theoretical value by the response surface on each Objective function, shown in Fig. 3. In Fig. 3(c,d), there is a spike so we analyzed the measurement value as the outlier based on the statistical analysis.

### Optimized solution search using MOGA

We validate the objective function that derives the nearest optimized solution to the actual radula. Single objective, two-objective, three-objective and four-objective optimization solutions each comprise _4_C_1_ = 4 ways, _4_C_2_ = 6 ways, _4_C_3_ = 4 ways and _4_C_4_ = 1 way. Sum of solutions is 15 ways in single optimization variation. The single-objective optimization solutions are listed in Tables 6 and 7. From Tables 6 and 7, the value of each single objective function was conflicted; thus, the radula was optimized for multi-objective optimization in Appendix 3.

**Table 6:** Single-objective optimization solution.

Objective function	Rake angle [deg.]	Round 1	Round 2	Tooth angle 1 [deg.]	Tooth angle 2 [deg.]	Tooth height [μm]
Radula shape	21.5	0.35	0.60	50.0	60.0	35.0
Cutting force minimization	44.6	0.70	0.70	29.1	28.1	53.4
Stress minimization	32.2	0.40	0.50	29.0	68.8	44.8
Volume minimization	—	0.80	0.80	10.0	10.0	15.0
Abrasion minimization	69.7	0.60	0.80	34.3	18.4	44.9

**Table 7:** Single-objective optimization solution.

Objective function	Cutting force minimization × 10^−5^ [N]	Stress minimization [MPa]	Abrasion minimization [Pa · μm/s]	Volume minimization (element counts)
Radula shape	6.06	2.49	19.9	15985
Single optimized solution value	1.38	1.84	3.00	327

## Discussion

### Comparison of analysis and estimated values

We analyzed the radula model by using finite element analysis. In this study, the radula would perform local grinding, one of the radula would perform cutting work, and grinding processing would be realized entirely by the entire radula. Therefore, we observed the local radula motion microscopically and estimated the total radula motion macroscopically. We used finite element analysis to compare the estimated and analysis values, and the values are listed in Table 8. The estimated vertical force on the radula was 1.33 N; the vertical force obtained in the radula shape analysis was 1.06 N. Therefore, the estimated and analysis values showed good correspondence. However, the tangential force obtained in the analysis was 5.81 N, but the estimated value was 3.31 N. The tangential force obtained in the analysis has some error. On the other hand, a comparison of the estimated value and that obtained in the cone shape analysis indicated that the cone shape matches the estimated shape. In the cone shape analysis, the tangential force was 3.27 N and the estimated value was 3.31 N. With respect to the vertical force, the analyzed value was 1.46 N and the estimated value was 1.33 N. These results show that the cone shape model would be suitable for analysis microscopically.

**Table 8:** Comparison between estimated and analyzed values on cone shape and radula shape models.

	Cutting force model ×10^−5^ [N]	Estimated value [N]	Cone shape analysis [N] (microscopically)	Radula shape analysis [N] (macroscopically)
Tangential	*f*_*t*_	3.31	3.27	5.81
Vertical	*f*_*n*_	1.33	1.46	1.06

### Comparison of optimized solution and radula shape

We investigated what design factors determined the radula shape. Table 9 shows a comparison of single objective optimization results. Table 9 lists the contradictory design variables among the objective functions. It indicates that the radula would have an optimized shape involving a few objective functions.

**Table 9:** Comparison of single-objective optimization results.

Objective function	Rake angle [deg]	Round 1	Round 2	Tooth angle 1 [deg]	Tooth angle 2 [deg]	Tooth height [μm]
Radula shape	22	0.60	0.40	50.0	60.0	35.0
Cutting force minimization	44.6	0.70	0.70	29.1	28.1	53.4
Stress minimization	32.2	0.40	0.50	29.0	68.8	44.8
Abrasion minimization	69.7	0.60	0.80	34.3	18.4	44.9
Volume minimization	—	0.80	0.80	10.0	10.0	15.0

Next, we studied the range of each objective function on all objective (four objectives) optimization. Table 10 lists the results of all objective function optimization. Table 11 shows that the cutting force was minimized by 36.3%, stress was minimized by 84.2%, abrasion was minimized by 56.6%, and volume was minimized by 7.31%. Especially, minimizing the stress by 84.2% considerably affected the shape of the radula. The results suggested that the shape of the radula would be optimized if stress and other objective functions are minimized. On the contrary, volume minimization would hardly influence the shape of the radula.

**Table 10:** Comparison between all objective optimization.

Objective function	Cutting minimization × 10^−5^ [N]	Stress minimization [MPa]	Abrasion minimization [Pa • μm/s]	Volume minimization (elements)
Radula shape	6.06	2.49	19.9	15985
Pareto solution minimization	1.38	1.92	30.9	730
Pareto solution maximization	7.35	3.62	38.8	16457
Minimization efficiency	36.3	84.2	56.6	7.31

**Table 11:** Radula shape.

Rake angle [deg.]	Round 1	Round 2	Tooth angle 1 [deg.]	Tooth angle 2 [deg.]	Tooth height [μm]
22	0.6	0.4	50	60	35

### Objective of optimization on radula

**Figure 4:**
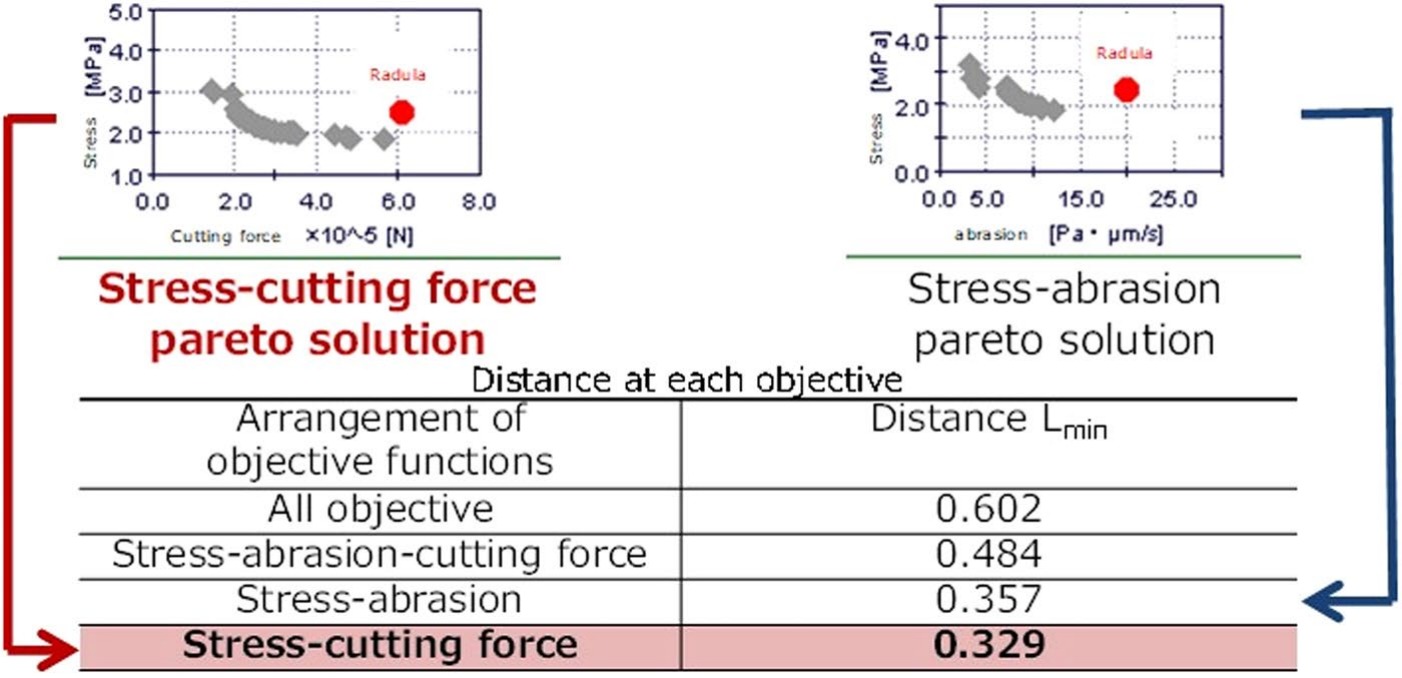
Comparison of distance between each objective function.

The range of pareto optimization is different for each objective function. To compare each solution, we normalized the solution so that the maximum value is 1 and the minimum value is 0. Next, to evaluate the optimization of each objective function, we calculated the distance L_min_ between the actual radula and the nearest normalized pareto solution. Figure 4 shows the distance between each arrangement of stress minimization and other objective functions. From Fig. 4, the lowest distance L_min_ is 0.329 for the stress–cutting force optimization. Thus, the solution of the stress–cutting force optimization is nearest to the performance of the actual radula. It indicates that the radula should be designed by optimizing the stress and cutting force.

In future works, we will try to the radula observation more precisely and model the radula using Hertzian-Mindlin solution. Furthermore, we will adapt the stress-cutting force objective optimization method to one with drilling functions such as the lathe. In addition, we will study not only radula, but also shell of Euhadra peliomphala. Since the shell of Euhadra peliomphala protect the soft bod from attacks like as Nacre^38–40^, we need clarify how the combination of shape and microstructure contribute to the mechanical robustness. Moreover, we would compare the mechanical properties and the objective function obtained from the radula shape of snail and shellfish with different food properties.

## Methods

### Modeling of grinding/cutting in Euhadra peliomphala

We observed the radula of Euhadra peliomphala. The radula was obtained using the following procedure.

1. Collect frozen alive Euhadra peliomphala.
2. Boil the frozen Euhadra peliomphala.
3. Take out the body 2.0–3.0 [mm] from the shell and cut it.
4. Open a part of the mouth and take out only the mouth.
5. Bath the mouth with a strongly basic drug to melt the meat.
6. Obtain the undissolved radula from the aqueous solution.

**Figure 5:**
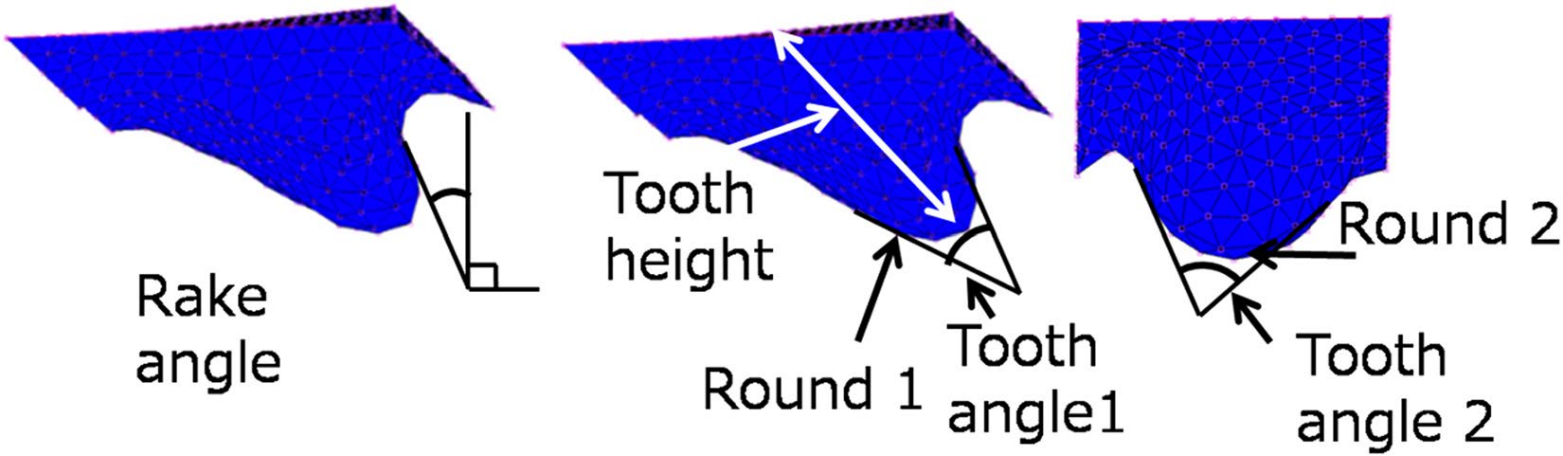
3D CAD model of radula shape.

We obtained the 3D shape of the radula by using a microscope (VHX-5000, KEYANCE) to determine the height data of each pixel. We developed a 3D CAD model from these height data, as shown in Fig. 5 and Table 11. Round 1 and Round 2 in Table 11 are defined by the rho dimension of a conic section.

**Figure 6:**
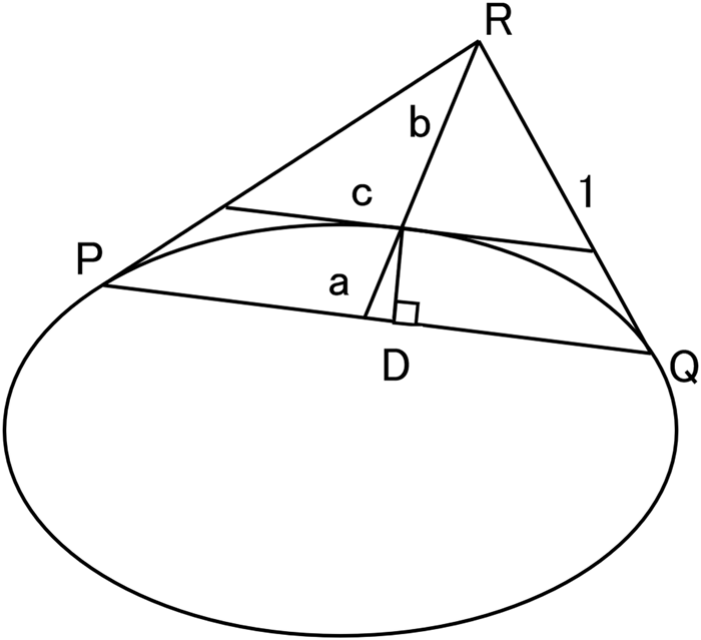
Rho dimension.

Round 1 and 2 are defined by the rho dimension of the conic curve. We describe rho in Fig. 6. The shape of the conic curve is defined by the rho dimension of the conical arc PQ. The straight-line segment PR is tangent to the ellipse at the point P and the straight-line segment QR is tangent to the ellipse at the point Q. The straight-line segment RD intersects the straight-line segment PQ at the point D, which is the midpoint of the straight segment PQ. The “rho” dimension is a value that specifies the ratio up to the point C to the vector up to the vertex R of the string PQ. Point C is the maximum normal distance (CD) from the string PQ to the conic arc PQ. The straight-line PR and the straight line QR are used for measuring the tooth angle.

In Table 11, tooth angle 2α is 60°. The coefficient of friction μ is 0.2, which is similar to the coefficient of friction between wood and wood because the Euhadra peliomphala has mucus in damp condition. We carried out an experiment and calculated the grinding energy ratio C_p_ [N/mm^2^]. It is defined as the energy required for removal per unit volume of food, in the Appendix. We investigated a food trace to decide the cutting depth Δ [mm] and grinding diameter of the radula D [μm] and observed the motion of Euhadra peliomphala by using a microscope to determine the effective tooth number N, the feeding speed ν [mm/s], the radula moving speed V [mm/s], and grinding width b [mm], as shown in Table 12.

**Table 12:** Model parameters.

Parameter	Symbol	Parameter	Symbol
Tangential grinding force N	*F*_*t*_	Vertical grinding force N	*F*_*n*_
Tangential cutting force per unit N	*F*_*t*_	Vertical cutting force per unit N	*F*_*n*_
Effective tooth number	N	Grinding energy ratio N/mm^2^	Cp
Feeding speed (Euhadra peliomphala moving speed) mm/s	*V*	Radula moving speed mm/s	V
Cutting depth mm	Δ	Grinding width (mouth width) mm	b
Tooth angle deg.	2α	Coefficient friction	μ
Grinding diameter of radula μm	D		

**Figure 7:**
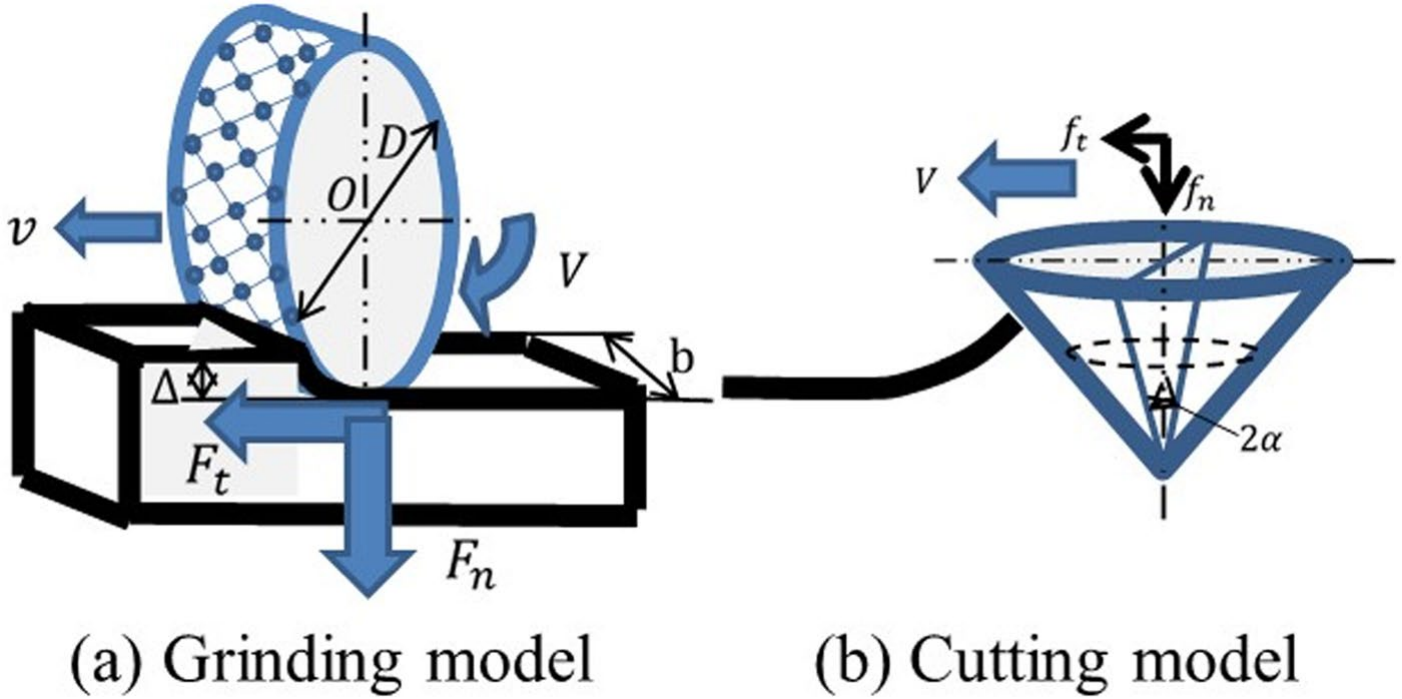
Grinding/cutting model of radula.

In this paper, we assume that a part of the radula is cut locally, but the overall radula grinds globally. Hertzian-Mindlin solution is a useful method which can know the outline of contact force separately from finite element analysis due to shape deformation at the time of contact^41^. On the other hand, obtaining the deformed shape is based on an analysis like the finite element method, and it is necessary to utilize the finite element method itself. For this reason, it is important to clarify whether Hertzian-Mindlin solution is applicable to the radula, but it will be a subject for the future. Based on the shape of radula, we develop the grinding and cutting model, as shown in Fig. 7 and Table 12. Thus, grinding force *F*_*t*_*, F*_*n*_ is determined by the effective tooth number N multiplied by the tangential cutting force per unit *f*_*t*_*, f*_*n*_. We determine the tangential grinding force *F*_*t*_ and vertical direction grinding force *F*_*n*_ by using equations (1) and (2).

1 $${F}_{t}=N\cdot {f}_{t}={C}_{p}(\frac{v\triangle b}{V})+\mu {F}_{n}$$

2 $${F}_{n}=N\cdot {f}_{n}={C}_{p}(\frac{\pi v{\rm{\Delta }}b}{2V})$$

From equations (1) and (2), equation (3) is calculated for determining Cp.

3 $$\frac{{F}_{t}}{{F}_{n}}={\rm{Cp}}(\frac{vb}{V})(\frac{{\rm{\Delta }}}{{F}_{n}})+\mu $$

Experiments were performed to determine the grinding energy ratio Cp, and the results are described in Appendix 1. The results of the Young’s modulus and Poisson’s ratio are described in Appendix 2.

### Optimization condition

We optimized the shape of the radula based on the L32 orthogonal table in Table 13 after 32 trials and developed the response surface for each objective function. We searched the optimized solution from the response surface by using multi-objective genetic algorithm, MOGA.

**Table 13:** L32 orthogonal table.

Number	Rake angle°	Round 1	Round 2	Tooth angle 1°	Tooth angle 2°	Tooth height mm
1	10	0.2	0.2	10	10	15
2	10	0.4	0.4	30	30	30
3	10	0.6	0.6	50	50	45
4	10	0.8	0.8	70	70	60
5	30	0.2	0.2	30	30	45
6	30	0.4	0.4	10	10	60
7	30	0.6	0.6	70	70	15
8	30	0.8	0.8	50	50	30
9	50	0.2	0.4	50	70	15
10	50	0.4	0.2	70	50	30
11	50	0.6	0.8	10	30	45
12	50	0.8	0.6	30	10	60
13	70	0.2	0.4	70	50	45
14	70	0.4	0.2	50	70	60
15	70	0.6	0.8	30	10	15
16	70	0.8	0.6	10	30	30
17	10	0.2	0.8	10	70	30
18	10	0.4	0.6	30	50	15
19	10	0.6	0.4	50	30	60
20	10	0.8	0.2	70	10	45
21	30	0.2	0.8	30	50	60
22	30	0.4	0.6	10	70	45
23	30	0.6	0.4	70	10	30
24	30	0.8	0.2	50	30	15
25	50	0.2	0.6	50	10	30
26	50	0.4	0.8	70	30	15
27	50	0.6	0.2	10	50	60
28	50	0.8	0.4	30	70	45
29	70	0.2	0.6	70	30	60
30	70	0.4	0.8	50	10	45
31	70	0.6	0.2	30	70	30
32	70	0.8	0.4	10	50	15

In this research, the reason why we used the MOGA method is because this method was performed based on multiple initial solutions, and it was used from the viewpoint that it is hard to arrive at a local solution due to occurrence of a mutation. The SOBOL sequence is used to obtain the initial generation (first generation). The SOBOL sequence is an experimental design method based on the quasi-random number method (SOBOL), which is suitable when the number of input variables is 2 to 20, and since the design is arranged more evenly than the uniform random number sequence, the multi-purpose GA is said to be suitable for generating the initial state of the first generation or MOGA.

Genetic algorithm (GA) is a learning algorithm that imitates the evolution of living things, which is very broad in scope. In other words, it genetically models genetic laws that have evolved over thousands of years, hundreds of millions of years, and yields a learning method that is useful for engineering design. GA flowchart is as follows.

1. Initialization: Generate N individuals with random chromosomes and set the initial generation population
2. Reproduction: Computation of each individual is calculated, and individuals are regenerated according to a certain rule depending on the goodness of fit. Here, some individuals with low fitness are culled out, and individuals with a high degree of adaptability will multiply as many as that number.
3. Crossover: Crossover is performed by the set crossover probability and crossover method to generate new individuals.
4. Mutation: Mutation is performed according to the set mutation probability and mutation method, and a new individual is generated. As a result, a population of a new generation is generated.
5. End judgment: If the termination condition is satisfied, the best individual obtained at that time is taken as the suboptimal solution of the problem. Otherwise return to step (2).

**Figure 8:**
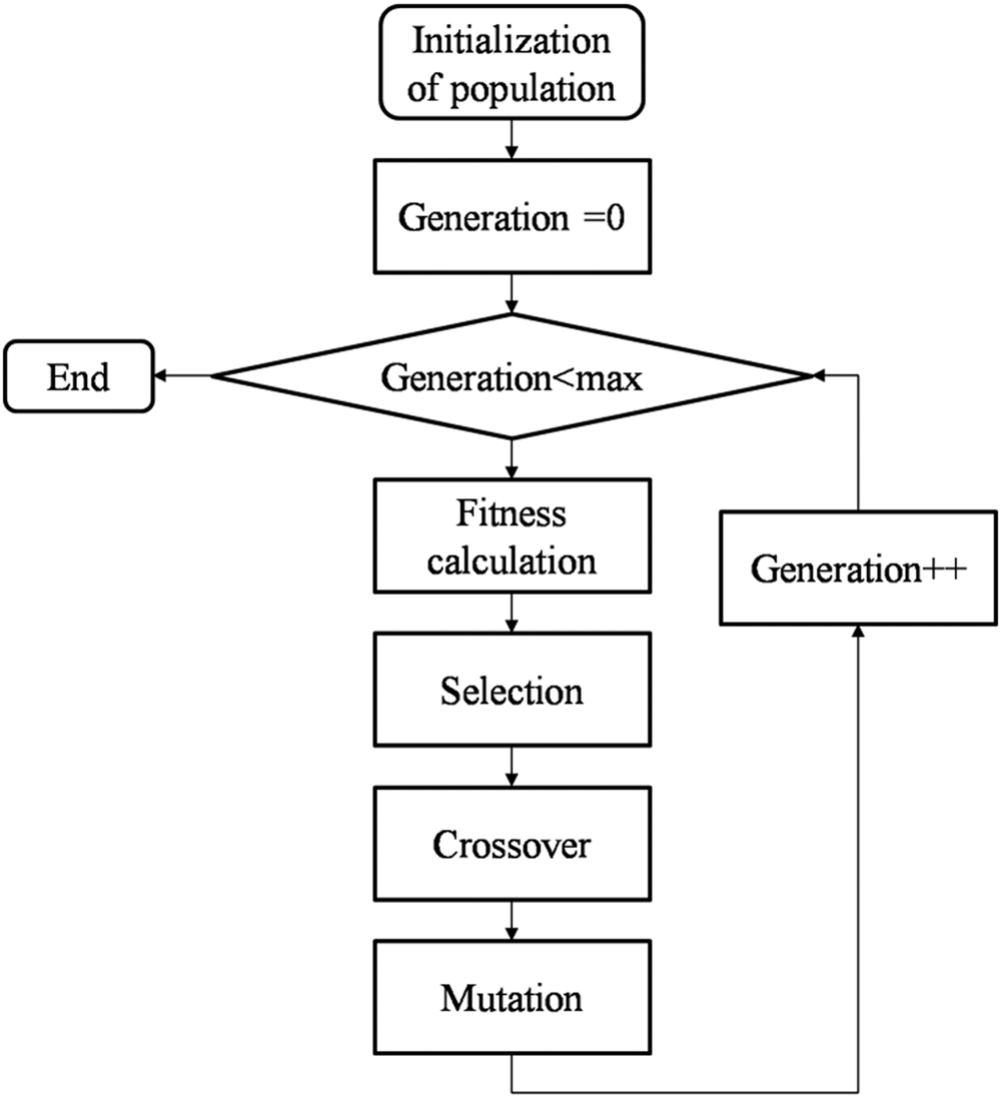
GA flowchart.

The basic operation of such a GA is represented by a flow chart as shown in Fig. 8. The end judgment condition in GA’s procedure (5) is one of the following three. One is that the maximum degree of conformity in the population exceeds the set threshold. Next is that the average fitness of the whole population exceeds the set threshold. Three is that the number of times of generation change exceeds a preset number of times. MPGA parameters are shown in Tables 14–17.

**Table 14:** Single objective optimization parameters.

Number of individuals per generation	16
Number of generations to evolve	20
Application probability of “directional crossover”	0.5
Application probability of “selection”	0.05
Application probability of “mutation”	0.1
Inversion rate of DNA by mutation	0.05
Elite strategy	Effectiveness
Handling of constraint condition	Adding penalty function to objective function
Algorithm type	MOGA- generation alternating function evolution
Seed for uniform random number generation	1

**Table 15:** Two objectives optimization parameters.

Number of individuals per generation	24
Number of generations to evolve	50
Application probability of “directional crossover”	0.5
Application probability of “selection”	0.05
Application probability of “mutation”	0.1
Inversion rate of DNA by mutation	0.05
Elite strategy	Effectiveness
Handling of constraint condition	Adding penalty function to objective function
Algorithm type	MOGA- generation alternating function evolution
Seed for uniform random number generation	1

**Table 16:** Three objectives optimization parameters.

Number of individuals per generation	24
Number of generations to evolve	50
Application probability of “directional crossover”	0.5
Application probability of “selection”	0.05
Application probability of “mutation”	0.1
Inversion rate of DNA by mutation	0.05
Elite strategy	Effectiveness
Handling of constraint condition	Adding penalty function to objective function
Algorithm type	MOGA- generation alternating function evolution
Seed for uniform random number generation	1

**Table 17:** All objectives optimization parameters.

Number of individuals per generation	60
Number of generations to evolve	100
Application probability of “directional crossover”	0.5
Application probability of “selection”	0.05
Application probability of “mutation”	0.1
Inversion rate of DNA by mutation	0.05
Elite strategy	Effectiveness
Handling of constraint condition	Adding penalty function to objective function
Algorithm type	MOGA- generation alternating function evolution
Seed for uniform random number generation	1

## Electronic supplementary material

Appendix
