# Supplementary material for: Design factors for determining the radula shape of Euhadra Peliomphala
Source: Sci Rep. 2019 Jan 24;9:749. doi: 10.1038/s41598-018-36397-x (PMC6345844; doi:10.1038/s41598-018-36397-x)
Supplement: Supplementary file 1 — Appendix [file 41598_2018_36397_MOESM1_ESM.pdf]

Design factors for determining the radula shape of Euhadra Peliomphala

Satoshi Miura[1*], Rikako Saito[1], Victor Parque[1] and Tomoyuki Miyashita[1]

Appendix

Appendix 1 Experiment for determining the value of Cp

We determined the grinding energy ratio Cp by using equations (1)– (3). After Euhadra Peliomphala ate the carrots, we measured the trace eaten on carrots to analyze the radula grinding and cutting force. The measurement results are listed in Tables 18 and 19 and Figure 9. From equation (3) and Figure 3, we determined the grinding energy ratio Cp 0.589 [N/mm^2].

Table 18 Measured vertical grinding and cutting forces

Displacement [mm]	Angle [rad.]	Fn [N]	Ft [N]
0.452	0.100	0.333	0.581
0.420	0.082	0.333	0.477
0.405	0.065	0.333	0.300
0.363	0.058	0.333	0.340
0.337	0.044	0.333	0.259
0.301	0.029	0.333	0.170
0.226	0.022	0.333	0.128
0.128	0.013	0.333	0.075

Table 19 Measurement parameters

Radula moving speed V [mm/s]	Feeding speed v [mm/s]	Grinding width b [mm]	Cutting depth Δ [mm]	Force ratio $\frac{F_t}{F_n}$	$\frac{vb\Delta}{VF_n}$
9329	300	30.00	0.217	1.747	1.080
9329	280	30.00	0.372	1.435	0.741
9329	290	30.00	0.274	0.902	0.556
9329	208	30.00	0.199	1.022	0.330
9329	253	30.00	0.163	0.777	0.335
9329	242	30.00	0.137	0.511	0.222
9329	206	30.00	0.095	0.386	0.159
9329	356	30.00	0.080	0.226	0.164

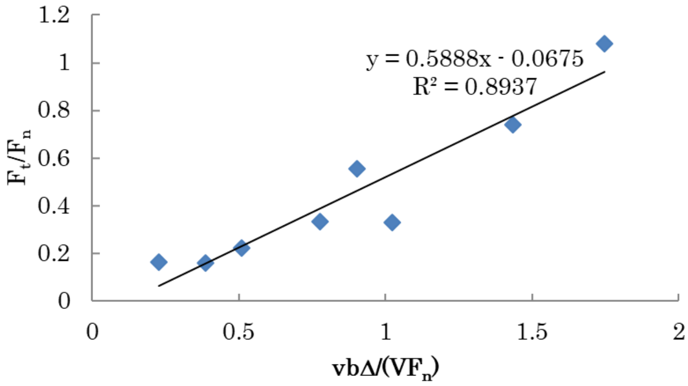

Figure 9 Results of grinding energy ratio Cp

Appendix 2 Young's modulus and Poisson's ratio

We simulated and analyzed the radula grinding model by using finite element analysis. In finite element analysis, each element connects each other initially on computer simulation. In finite element analysis, when loaded, the inter-element connection was released according to the elastic-plastic properties of the material, again performing element division and continuing the elasto-plastic analysis.

It is impossible to measure the material constant of the radula. The nano-indentation method is generally used for nanoscale measurement; however, it is necessary to maintain strict drying and temperature conditions. Therefore, the nano-indentation method is not appropriate as the radula is always wet. The radula is composed of diner, protein, and calcium carbonate; therefore, we mixed these materials. Compared with the experimental cutting force value, Young's modulus and Poisson' ratio of the radula were determined. Table 20 lists the values of the Young's modulus and Poisson's ratio obtained in the analysis.

Table 20 Material constant search analysis condition

Number	Young's modulus [GPa]	Poisson's ratio
1	5	0.3
2	5	0.4
3	10	0.3
4	10	0.4
5	15	0.3
6	15	0.4
7	20	0.3
8	20	0.4
9	25	0.3
10	25	0.4
11	30	0.3
12	30	0.4
13	35	0.3
14	35	0.4
15	40	0.3
16	40	0.4

Figures 10 and 11 and Table 21 show the results of the tangential and vertical cutting forces for each value of Young's modulus and Poisson's ratio.

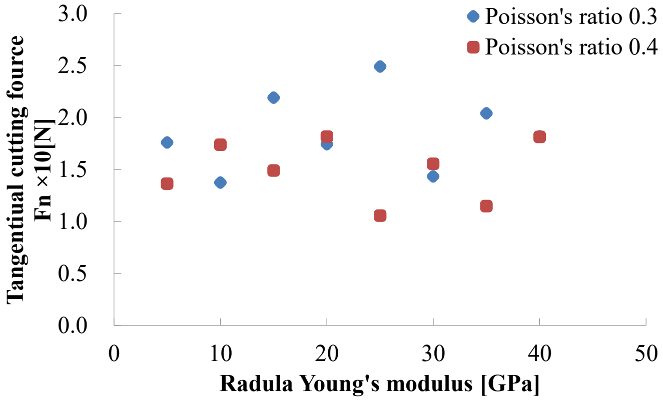

Figure 10 Tangential cutting vs. Radula Young's modulus

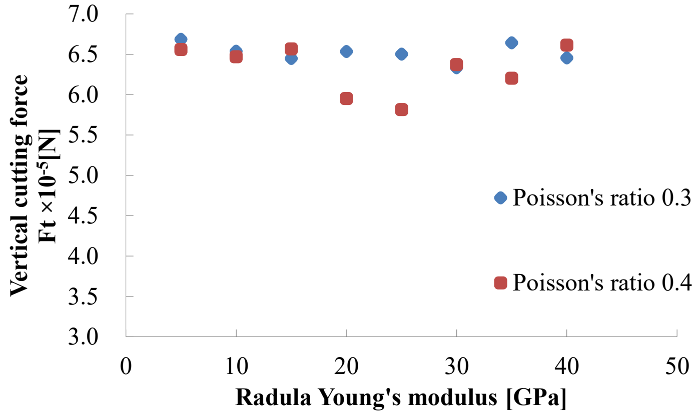

Figure 11 Vertical cutting force vs. Young's modulus of the radula

Table 21 Analysis results of radula simulation

Young's modulus [GPa]	Poisson's ratio 0.3		Poisson's ratio 0.4	
	$f_t \times 10^{-5}$ [N]	$f_n \times 10^{-5}$ [N]	$f_t \times 10^{-5}$ [N]	$f_n \times 10^{-5}$ [N]
5	6.69	1.76	6.56	1.37
10	6.54	1.37	6.47	1.74
15	6.45	2.19	6.56	1.49
20	6.53	1.74	5.95	1.82
25	6.50	2.49	5.81	1.06
30	6.33	1.43	6.37	1.55
35	6.64	2.04	6.20	1.15
40	6.45	1.82	6.61	1.81

The tangential cutting force f_t was 3.31×10^{-5} N and the vertical cutting force f_n as 1.33×10^{-5} N in the experiments. The tangential cutting force f_t was 5.81×10^{-5} N and the vertical cutting force f_n was 1.06×10^{-5}N in the analysis. The analysis results are similar to the experimental results at a Young's modulus of 25.0 [GPa] and Poisson's ratio of 0.4]. Figures 12–14 show the finite element analysis results of the radula using MARC (MSC Software, USA).

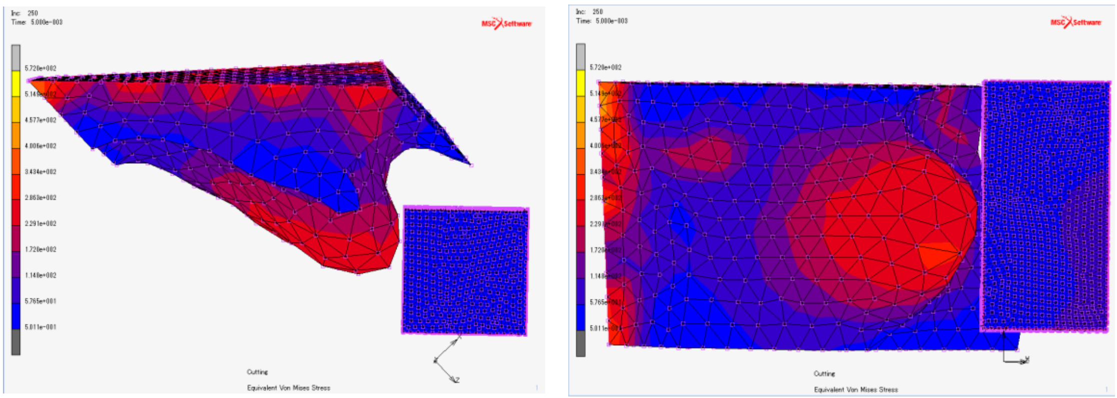

(a) Side view (b) Top view

Figure 12 Maximum stress

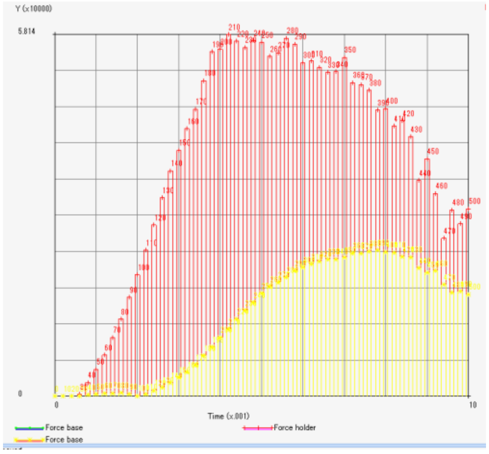

Figure 13 Longitudinal cutting force

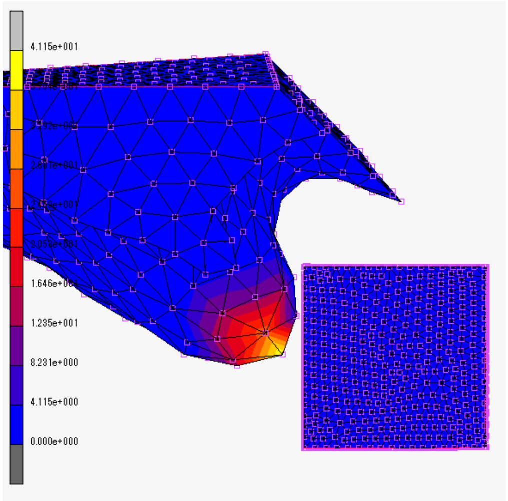

Figure 14 Polishing of radula in finite element analysis

Appendix 3 Pareto solution for multi-objective optimization

We show the pareto solution for the multi-objective optimization solution. Figures are the pareto solution for two (Figure 15-20), three (Figure 21-24) and four(all) (Figure 25)-objective optimization solution because two-objective, three-objective and four-objective optimization solutions each comprise ${}_4C_2$=6 ways, ${}_4C_3$=4 ways and ${}_4C_4$=1 way.

Two-objective optimization solution

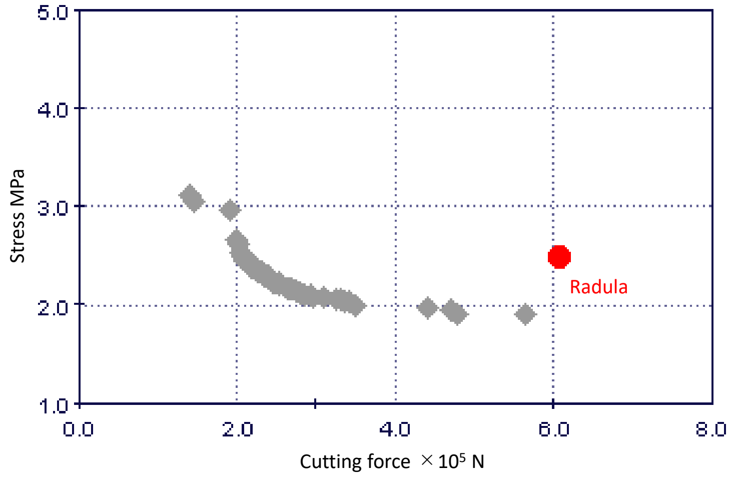

Figure 15 Stress–cutting force pareto solution

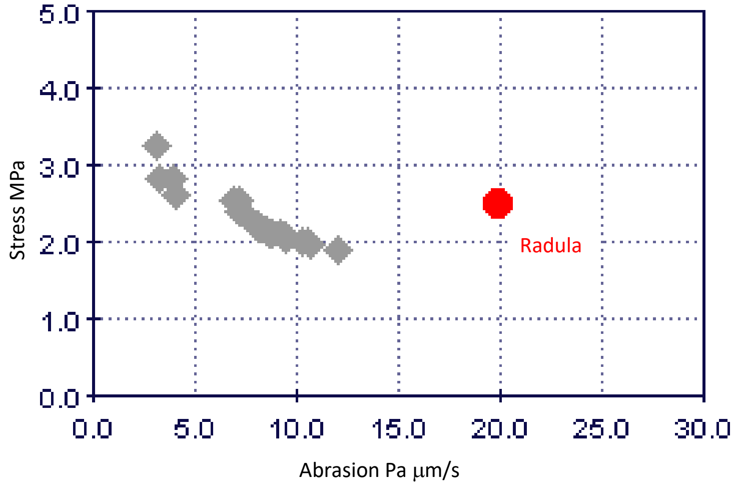

Figure 16 Stress–abrasion pareto solution

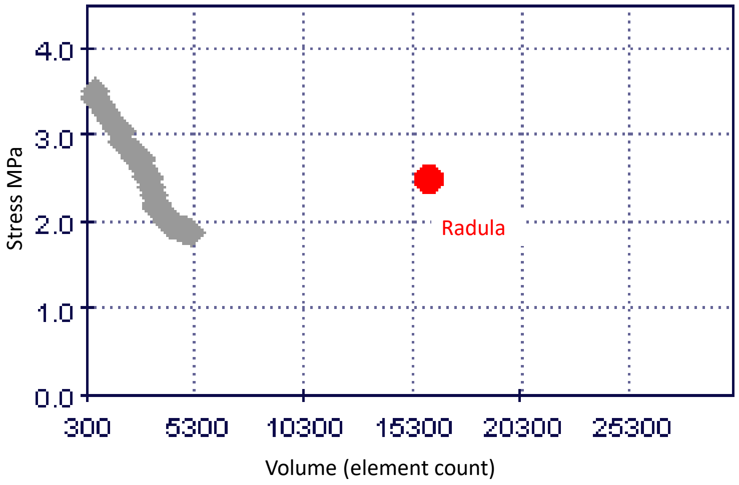

Figure 17 Stress–volume pareto solution

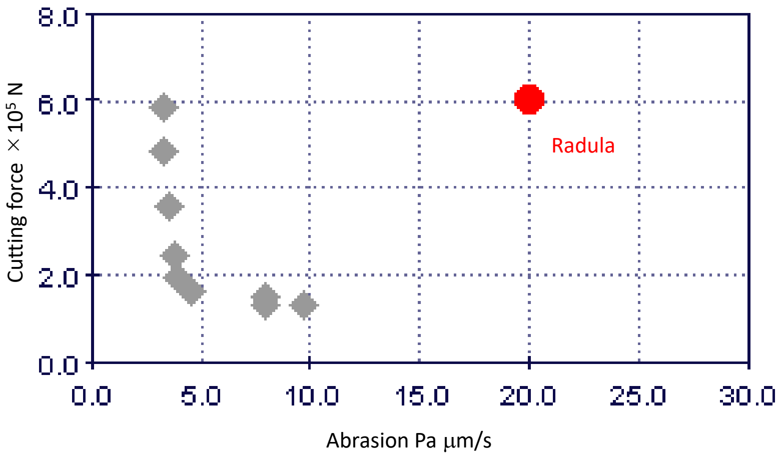

Figure 18 Cutting force–abrasion pareto solution

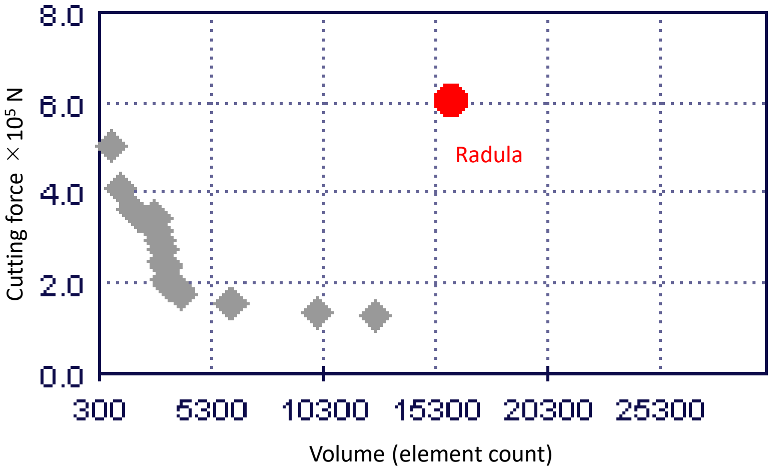

Figure 19 Cutting force–volume pareto solution

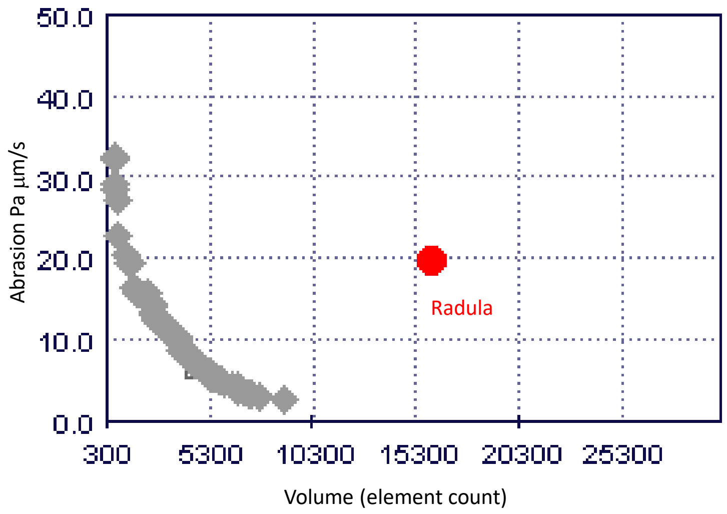

Figure 20 Abrasion–volume pareto solution

Three-objective optimization solution

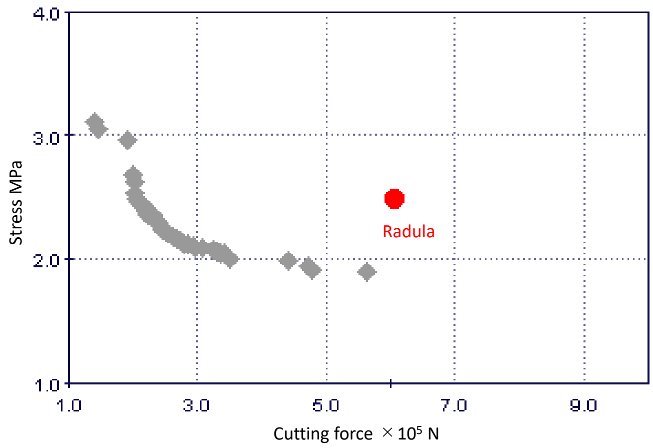

(a) Stress–cutting force

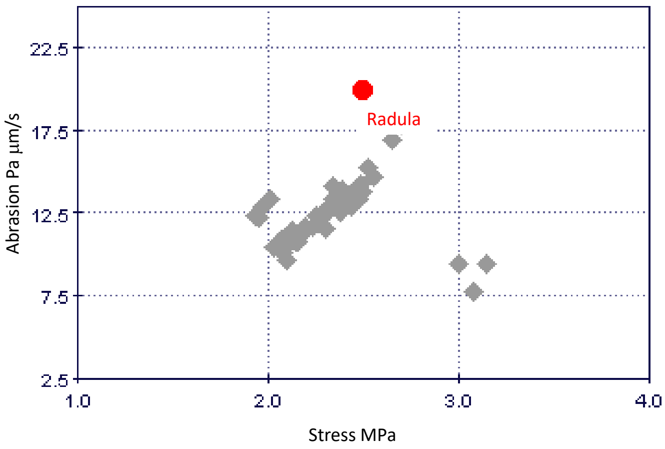

(b) Abrasion–stress

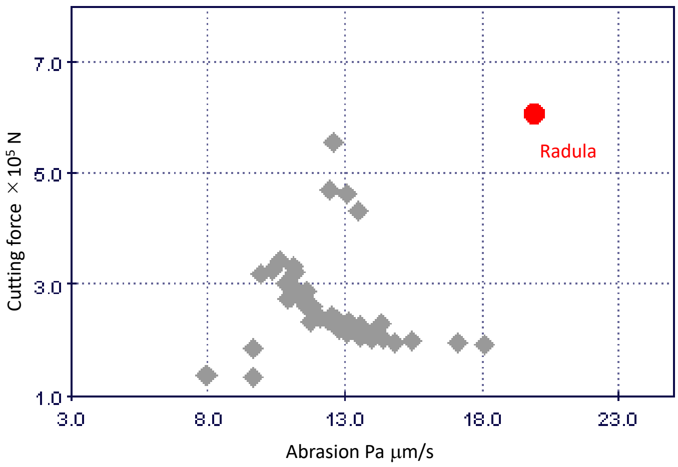

(c) Cutting force–abrasion

Figure 21 Cutting force–stress–abrasion pareto solution

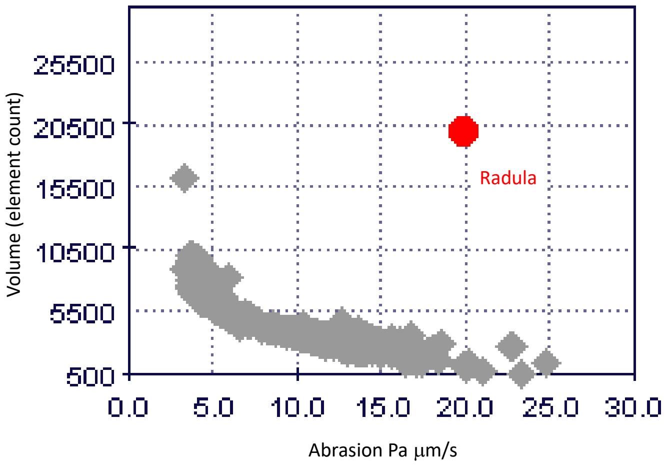

(a) Abrasion–volume

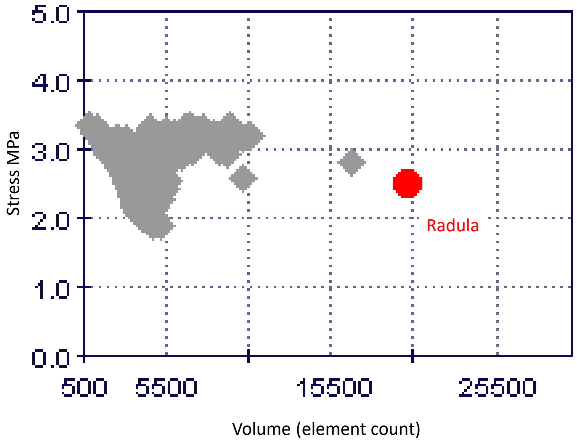

(b) Abrasion–stress

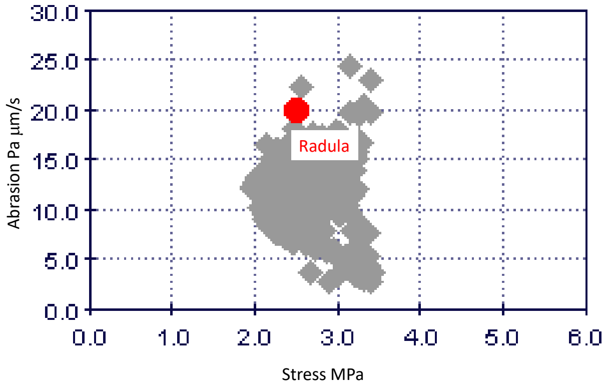

(c) Stress–volume

Figure 22 Stress–abrasion–volume pareto solution

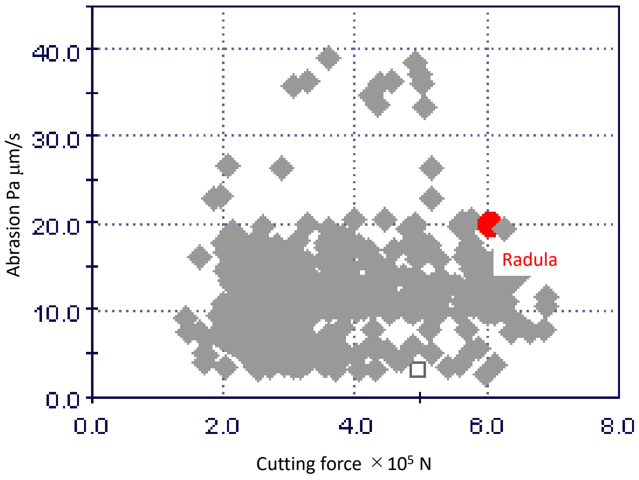

(a) Abrasion–cutting force

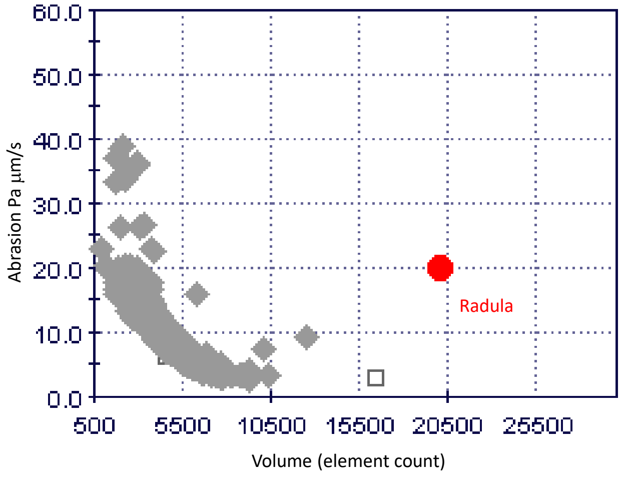

(b) Abrasion–volume

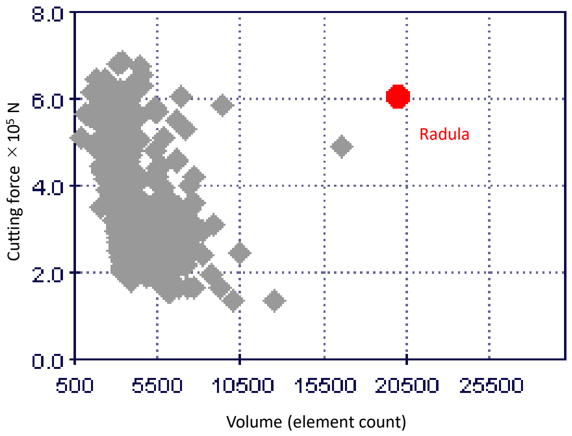

(c) Cutting force–volume

Figure 23 Abrasion–volume–cutting force pareto solution

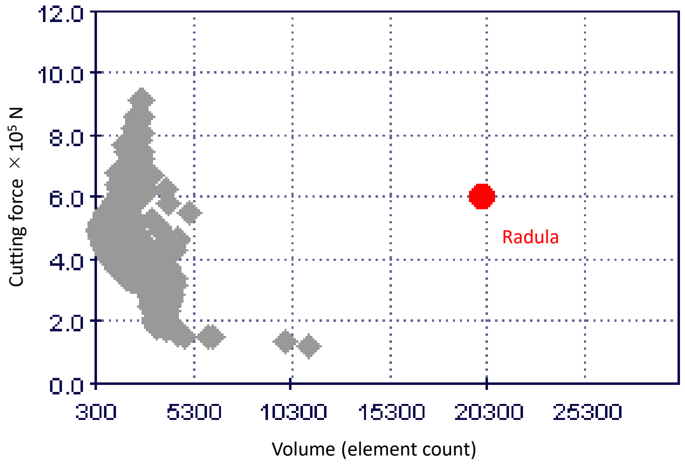

(a) Cutting force–volume

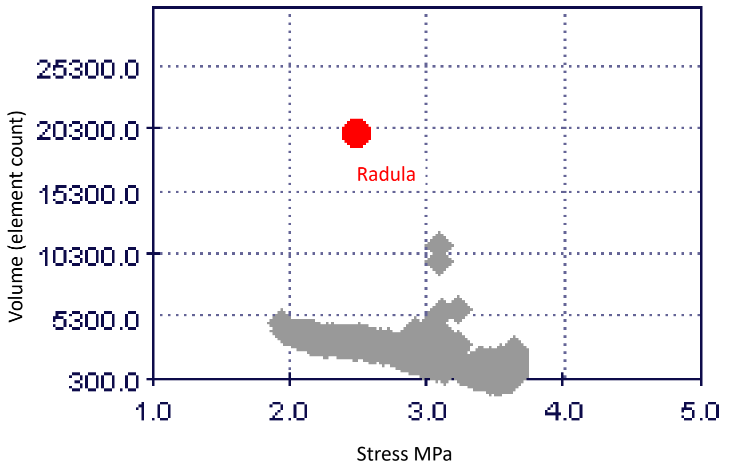

(b) Volume–stress

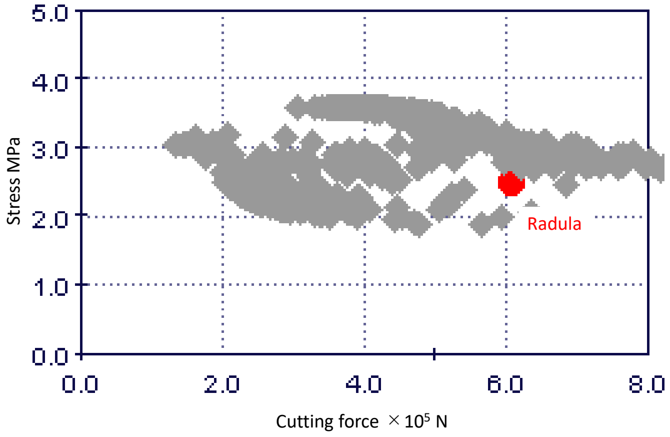

(c) Stress–cutting force

Figure 24 Stress–volume–cutting force pareto solution

Four(all)-objective optimization solution

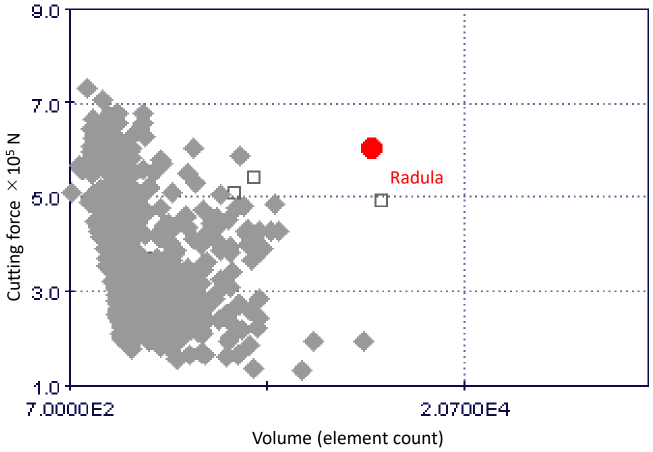

(a) Cutting force–volume

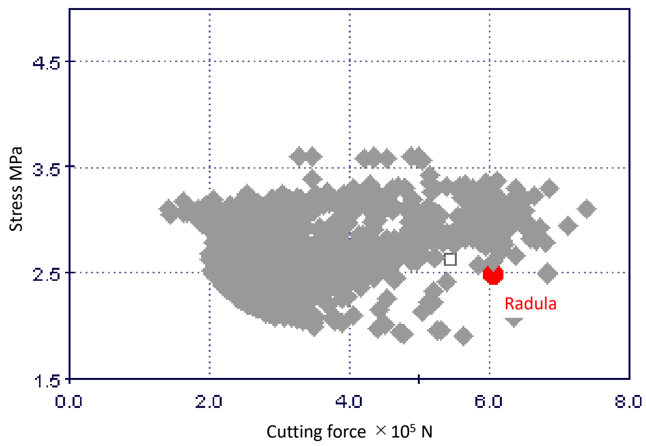

(b) Stress–cutting force

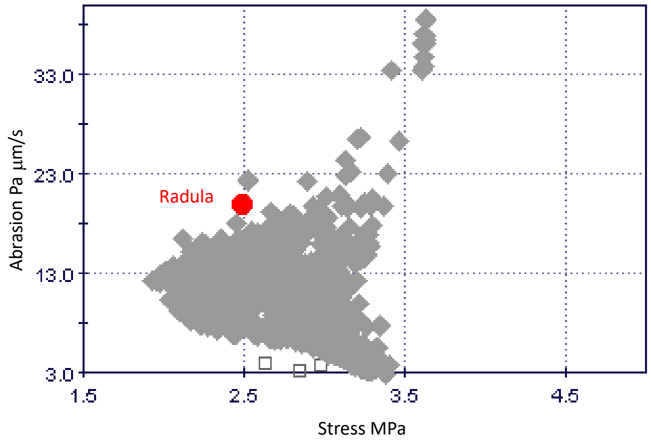

(c) Abrasion–stress

Figure 25 Four-objective optimization pareto solution
